# Heparin does not improve myocardial glucose metabolism suppression in [18 F]FDG PET/CT in patients with low β-hydroxybutyrate level

**DOI:** 10.1186/s13550-024-01153-y

**Published:** 2024-10-08

**Authors:** Suvi Hartikainen, Ville Vepsäläinen, Tiina Laitinen, Marja Hedman, Tomi Laitinen, Tuomo Tompuri

**Affiliations:** 1 Department of Clinical Physiology and Nuclear Medicine, Kuopio University Hospital, Kuopio, Finland; 2 Institute of Clinical Medicine, University of Eastern Finland, Kuopio, Finland; 3 Heart Centre, Kuopio University Hospital, Kuopio, Finland; 4 Heart Hospital, Tampere, Finland; 5 Department of Clinical Physiology, North Karelia Central Hospital, Joensuu, Finland

**Keywords:** Myocardial inflammation, Beta-hydroxybutyrate, BHB, FDG-PET, Glucose metabolism, Myocardium, Heparin

## Abstract

**Background:**

Inadequate myocardial glucose metabolism suppression (GMS) can hamper interpretation of cardiac [^18^F]fluorodeoxyglucose (FDG) positron emission tomography (PET/CT). Use of β-hydroxybutyrate (BHB) measurement before [^18^F]FDG injection has been proposed for predicting adequate GMS. However, limited information is available on BHB measurement in guiding preparations for [^18^F]FDG-PET/CT. The purpose of this study was **t**o evaluate if point-of-care measured BHB is useful in guiding heparin premedication for cardiac [^18^F]FDG-PET/CT.

**Results:**

155 patients (82 male) had followed a high-fat, low-carbohydrate diet and fasted for at least twelve hours. For the first 63 patients, BHB was measured, but it was not used to guide premedication. For the subsequent 92 patients, heparin 50 IU/kg was injected intravenously 15–20 min before [^18^F]FDG injection if the BHB level was low (< 0.35 mmol/l). Cardiac [^18^F]FDG uptake pattern was evaluated visually and [^18^F]FDG uptake in the myocardium and blood pool were measured. Median BHB level was 0.4 (range 0.1–5.8) mmol/l. Eighty-eight patients (57%) reached a BHB level higher than 0.35 mmol/l. 112 patients (72%) had adequate GMS. In the high BHB group, 74 patients (84%) had adequate GMS, whereas of those with low BHB, only 38 (57%) had adequate GMS (*p* < 0.001). In the low BHB group, the prevalence of inadequate GMS was comparable in patients with and without heparin (44% vs. 42%, *p* = 0.875).

**Conclusions:**

While high BHB predicts adequate GMS, unfractionated heparin does not improve GMS in patients with low BHB.

## Background

[^18^F]fluorodeoxyglucose ([^18^F]FDG) positron emission tomography (PET/CT) is used for diagnosing and monitoring inflammatory cardiac diseases. To suppress physiological uptake, the European Association for Cardiovascular Imaging and European Association for Nuclear Medicine guidelines, as well as the Society for Nuclear Medicine and Molecular Imaging and the American Society for Nuclear Cardiology expert consensus document recommend a high-fat diet lacking carbohydrates for 12–24 h combined with fasting for 12–18 hours before [^18^F]FDG injection [1, 2]. Still, inadequate suppression may hamper the diagnosis or exclusion of inflammatory cardiac diseases [3, 4].

Unfractionated heparin is suggested to further suppress physiological uptake [3, 4]. Heparin activates lipoprotein lipase and can thus be expected to boost the effects of the ketogenic diet. Meta-analysis of different preparation methods shows that while heparin can boost the effects of the ketogenic diet, it does not replace it [5].

Studies on β-hydroxybutyrate (BHB) to predict adequate suppression of myocardial glucose uptake have been reported recently [6–8]. However, there is no agreement on what should be done regarding patients with low BHB levels.

The purpose of our study was to assess whether BHB measurement could be used to guide heparin use in cardiac [^18^F]FDG PET/CT.

## Methods

### Patient preparation

Between November 2021 and June 2024, 156 patients with a clinical indication of known or suspected inflammatory cardiac disease for cardiac [^18^F]FDG PET/CT were recruited. Patients were not recruited if intravenous unfractionated heparin could not be used, i.e., patients on anticoagulation therapy or with significant risk for bleeding. Patients received diet instructions in a letter detailing foods that were allowed as well as those that would not be allowed, and instructions to keep a food diary. Upon arriving at the imaging centre the patients’ diary was checked. Only patients who were deemed compliant with ketogenic diet after checking food diary were recruited. All patients had been on a low-carbohydrate, high-fat diet for 1–2 days and fasting for at least 12 h before [^18^F]FDG injection. Weight and height were measured. Blood glucose level was measured with Accu-Chek Performa (Roche Diagnostics GmbH, Mannheim, Germany), and BHB level with Freestyle Precision NEO (Abbott Diabetes Care Ltd., Witney, Oxon, UK) point-of-care devices. Heparin was not used for the first 63 patients. If the point-of-care BHB level was less than 0.35 mmol/l [8], the subsequential 92 patients received unfractionated heparin 50 IU/kg intravenously. One patient received heparin with BHB > 0.35 mmol/l and was excluded from the study, leaving 155 data sets.

After at least 30 min of rest, an [^18^F]FDG dose of 3 MBq/kg was administered intravenously 60 min before the scan while resting continued.

### Data acquisition

All patients were scanned using a Biograph Vision PET/CT (Siemens Healthineers, Knoxville, TN, USA) system. Iterative reconstruction with four iterations with time-of-flight (TOF) and point spread function (PSF) was used for image reconstruction. The image data was analyzed using Hybrid Viewer (Hermes Medical Solutions, Stockholm, Sweden). Images were tilted according to cardiac planes for cardiac [^18^F]FDG uptake measurement. Four circular regions of interest (ROIs) with 1 cm diameter were drawn in the ventricular septum, four in the lateral wall, and one in the left chamber blood pool using the four-chamber view. In the case of inflammatory cardiac diseases, focal uptake areas were excluded from the analysis. Myocardial uptake was measured as the maximum standardized uptake value normalized by calculated lean body mass (SULmax). The mean of SULmax values of the left ventricle myocardial ROIs was calculated (SUL_LV_). SUL_LV_ ratio to left ventricle blood pool background (SULratio) was calculated, and SULratio ≤ 1 was considered adequate myocardial suppression. Visual assessment of uptake was classified as ‘none’, ‘focal’, ‘diffuse’, ‘focal on diffuse’, ‘basal ring’ or ’lateral wall uptake’. ‘None’ and ‘focal’ were classified as adequate suppression.

### Statistical analysis

IBM SPSS Statistics (version 29.0.1.0, IBM Corp., Armonk, NY, USA) was used for the statistical analysis. Continuous variables are represented as median values with minimum and maximum values, and categorical variables are represented as counts with percentages. Spearman’s ρ was used to evaluate bivariate correlations between SUV_LV_ values and other variables. Continuous variables were tested using the Mann-Whitney U test, and categorical variables using the Pearson χ^2^ test. Correlations between SULratio and BHB level were calculated using Spearman’s ρ, and the significance of the difference in correlations was computed using cumulative distribution function. Receiver operating characteristic (ROC) curve analysis was used to evaluate to ability of BHB level to predict adequate suppression. A *p*-value of 0.05 was considered statistically significant. Logarithmic correction was performed on SULratio for interaction analysis. ANCOVA interaction analysis was performed to assess possible interaction of sex and heparin on SULratio.

## Results

### Patient characteristics

Characteristics of patients are shown in Table 1. The median (min-max) age of the study population was 58 (19–79) years. The median blood BHB level was 0.4 (0.1–5.8) mmol/l. Using a cut-off of 0.35 mmol/l, 67 patients (43%) had low BHB level. Of those with low BHB, 40 received unfractionated heparin. The median SULratio was 0.80 (0.45–12.94). In semiquantitative assessment myocardial suppression was adequate for 112 patients (72%). One hundred forty-nine patients had followed a ketogenic diet for one day, and of those, 84 (56%) had BHB higher than 0.35 mmol/l. Six patients had followed the diet for two days.

The median body mass index (BMI) was 28.3 (17.0-54.4) kg/m^2^. Of the whole study population, 61 (39%) were obese (BMI ≥ 30 kg/m^2^).

In visual assessment, 88 patients had no uptake, 22 had focal uptake, nine had isolated lateral wall uptake, six had basal ring type uptake, 16 had diffuse uptake and fourteen had focal on diffuse uptake.

**Table 1:** Patient characteristics

	Complete cohort (*n* = 155)	Inadequate suppression (*n* = 43)	Adequate suppression (*n* = 112)	*p*
Age (years)	58 (19–79)	59 (39–76)	58 (19–79)	0.480
Glucose (mmol/l)	5.0 (2.8–10.4)	5.1 (3.5–6.8)	5.0 (2.8–10.4)	0.623
BHB (mmol/l)	0.4 (0.1–5.8)	0.3 (0.1–2.6)	0.4 (0.1–5.8)	**< 0.001**
Weight (kg)	84 (49–140)	83 (54–117)	84 (49–140)	0.457
BMI (kg/m^2^)	28.3 (17.0–54.4)	27.9 (19.4–40.0)	28.6 (17.0–54.4)	0.548
SUL_LV_	1.20 (0.66–13.72)	2.11 (1.18–13.72)	1.12 (0.66–1.66)	**< 0.001**
SULratio	0.80 (0.45–12.94)	1.36 (1.01–12.94)	0.70 (0.45–1.00)	**< 0.001**
Male	82 (53%)	22 (51%)	60 (55%)	0.788
Diabetes	13 (8%)	1 (2%)	12 (11%)	0.092
Obese*	61 (39%)	16 (37%)	45 (40%)	0.735
BHB higher than 0.35 mmol/l	88 (57%)	14 (33%)	74 (66%)	**< 0.001**

Values are presented as medians (range) for continuous variables and counts (percentages) for discrete variables. *p* < 0.05 is considered significant (bolded). *BHB*, beta-hydroxybutyrate; *BMI*, body mass index; *SUL*, standardized uptake value using calculated lean body mass. *Using WHO classification, BMI ≥ 30 kg/m^2^

### Comparison of women and men

Of the study population (*n* = 155), 82 (53%) were male. Blood glucose level was 4.9 (2.8–7.4) mmol/l for females and 5.2 (3.6–10.4) mmol/l for males (*p* = 0.022), and BHB level 0.4 (0.1-3.0) mmol/l for females and 0.4 (0.1–5.8) mmol/l males (*p* = 0.170). There were no other statistically significant differences between female and male groups. In interaction analysis, there was no interaction between sex and heparin on SULratio (*p* = 0.573). Furthermore, there was no difference between the correlation of SULratio and BHB level (-0.390 for males, -0.354 for females, *p* = 0.799) or the area under the ROC curve (0.697 for males, 0.704 for females, *p* = 0.943) between the sexes.

### Comparison of high and low BHB groups and heparin and no-heparin groups

A comparison of high and low BHB groups, as well as of heparin and no-heparin groups with low BHB levels, can be seen in Table 2. Those with high BHB had significantly lower SULratio (mean 0.70) and a higher proportion of adequate suppression (84%) than those with low BHB level (0.95 and 57%, respectively, *p* < 0.001 for both variables). Patients with high BHB level also had significantly lower blood glucose than patients with low BHB level (4.9 mmol/l and 5.2 mmol/l, *p* = 0.001).

**Table 2:** Comparison of patients with high and low BHB, and no-heparin and heparin groups

	Complete cohort (*n* = 155)			Low BHB (*n* = 67)		
	High BHB (*n* = 88)	Low BHB (*n* = 67)	*p*	No heparin (*n* = 27)	Heparin (*n* = 40)	*p*
Age (years)	58 (23–79)	56 (19–75)	0.176	56 (19–75)	56 (32–74)	0.969
Glucose (mmol/l)	4.9 (2.8–10.4)	5.2 (4.2–9.4)	**0.001**	5.3 (4.2–9.4)	5.2 (4.2-7.0)	0.473
Weight (kg)	82 (49–138)	87 (57–140)	**0.014**	86 (63–140)	89 (57–133)	0.274
BMI (kg/m^2^)	28.1 (17.0-54.4)	29.4 (19.4–47.9)	0.053	29.4 (22.5–47.9)	29.2 (19.4–43.9)	0.227
SULratio	0.70 (0.45–3.30)	0.95 (0.57–12.94)	**< 0.001**	0.96 (0.65–3.51)	0.92 (0.57–12.94)	0.808
Male	43 (49%)	39 (58%)	0.248	12 (44%)	25 (68%)	0.061
Diabetes	9 (10%)	4 (6%)	0.344	1 (4%)	3 (8%)	0.520
Obese*	30 (34%)	31 (46%)	0.124	13 (48%)	18 (45%)	0.800
Adequate suppression	74 (84%)	38 (57%)	**< 0.001**	15 (56%)	23 (58%)	0.875

Values are presented as medians (range) for continuous variables and counts (percentages) for discrete variables. *p* < 0.05 is considered significant (bolded). BHB, beta-hydroxybutyrate; BMI, body mass index; SUL, standardized uptake value using calculated lean body mass. *Using WHO classification, BMI ≥ 30 kg/m^2^

**Fig. 1:**
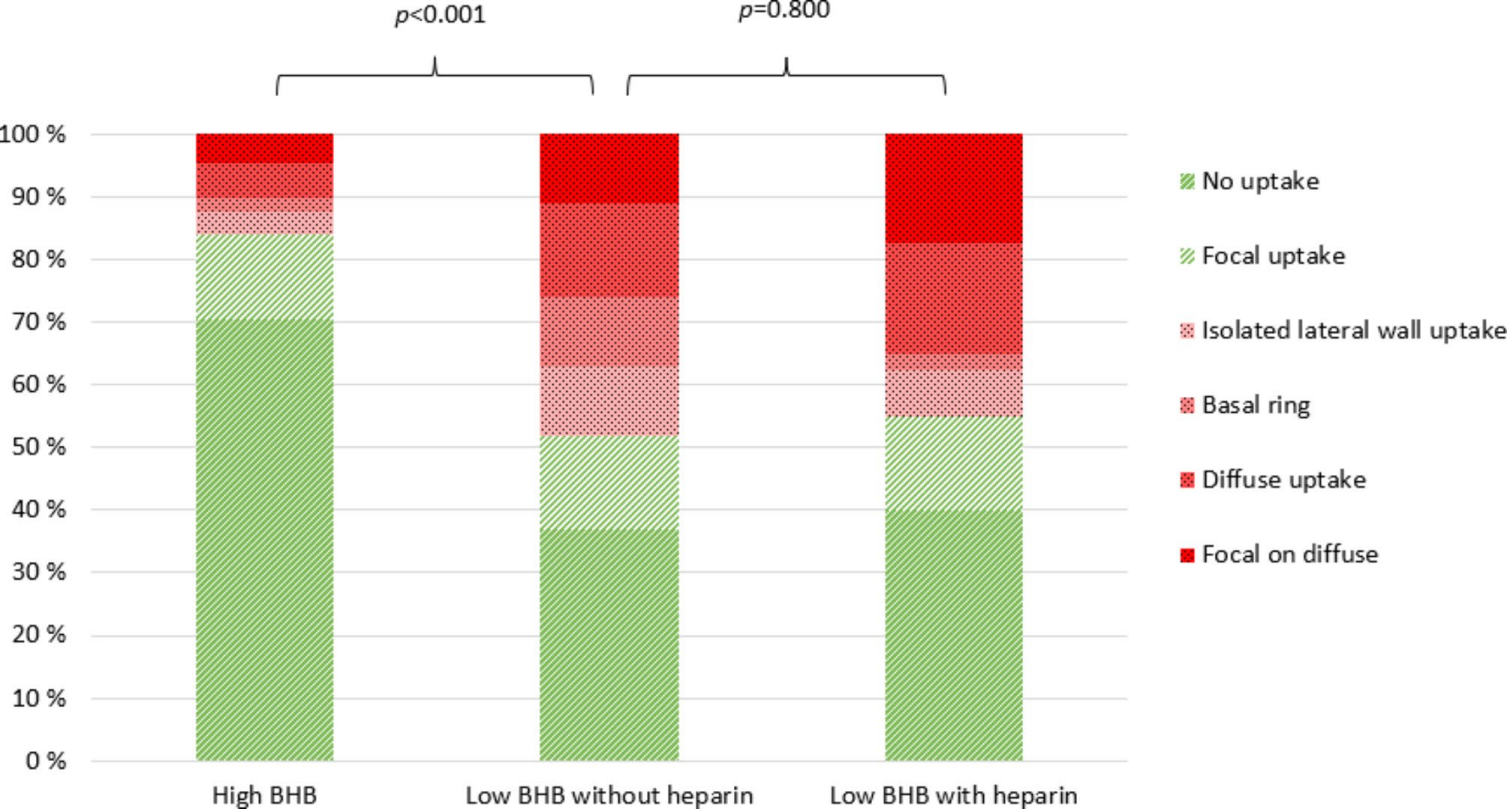
Visual uptake pattern distribution in groups. Green, adequate suppression; red, inadequate suppression. BHB, β-hydroxybutyrate level. High BHB, BHB > 0.35 mmol/l; low BHB, BHB < 0.35 mmol/l

There was no statistically significant difference in the myocardial suppression between the heparin and the no-heparin groups in the semiquantitative or visual assessment (Fig. 1), nor were there any other statistically significant differences between the two groups. When only those with no uptake were considered as adequate suppression, the results remained practically the same (data not shown).

## Discussion

Limited data exists on the impact of heparin administration on the image quality in cardiac [^18^F]FDG-PET/CT in patients with low BHB levels. We aimed to explore this issue in a consecutive patient population, comparing patients with and without heparin premedication. Our study confirms that while the BHB level is high, the percentage of adequate suppression is high. It also shows that while the BHB level is low, the percentage of adequate suppression is significantly lower, and unfractionated heparin does not seem to improve myocardial suppression in that group significantly. Rather, a high BHB level should be pursued. However, as many as 45% of patients had a low BHB level after a one-day diet. Thus, we suggest that a longer diet is needed to promote more prominent ketosis and better myocardial suppression.

Both unfractionated and low-molecular-weight, heparin increases hepatic lipase and lipoprotein lipase mass and activity, but the effect of low-molecular-weight heparin is less prominent due to faster clearance of lipoprotein lipase by the liver [9]. Thus, heparin raises free fatty acid and ketone body levels [10]. Considering our results, this effect does not seem marked enough to tilt the odds of suppression in patients with a low initial BHB level.

Though studies are reporting the use of heparin premedication for [18 F]FDG PET/CT as early as 2005, only a few published, controlled studies report the glucose metabolism suppressing effects of heparin [3, 4, 11–16]. Morooka et al. reported in 2014 that 18 h of fasting is more efficient than heparin combined with 12 h of fasting in suppressing myocardial glucose metabolism [14]. Masuda and Scholtens reported in 2016 that heparin combined with standard preparation (either 18 h of fasting in Masuda’s study or ketogenic diet in Scholtens’s study) is superior to standard preparation alone [4, 15]. Giorgetti et al. showed in 2017 that warfarin, compared to heparin, has a lower but significant effect on myocardial glucose metabolism [16]. Scholtens later published the impact of heparin dose variation on the level of GMS, stating that 50 IU/kg and not 15 IU/kg is the effective dose [3]. However, Larson et al. reported their preparation protocol with a ketogenic diet starting 36 h before the scan and three small heparin doses of 10 IU/kg, two of which were injected after the radiopharmaceutical [17]. They reported that the protocol leads to very low amount of non-diagnostic scans, with only 6% of scans having either diffuse or focal-on-diffuse pattern. Yet, there were no control subjects in that study, so it is unknown whether low level of unsatisfactory suppression is due to heparin, the elongated diet, or the combination of those.

Williams and Kolodny were the first to report superior suppression of very low carbohydrate, high-fat meals compared to overnight fasting [18]. Since then, the diet has been applied before fasting, and the duration has gained length. Current guidelines recommend dieting 24 h before [^18^F]FDG injection and fasting at least 12 h before injection. However, Lu et al. reported in 2017 that a 72-hour-diet is far superior to a 24-hour-diet [19]. Similarly, Özütemiz et al. reported in 2021 that a 72-hour ketogenic diet with overnight fasting every three nights is more efficient than a 24-hour diet with 12 h of fasting, although the shorter diet also allowed vegetables while the longer diet did not [20]. In our centre, vegetables are currently not allowed in the ketogenic diet, because the amount of carbohydrates is not easily monitored. However, this may contribute to gastrointestinal side effects. Further research is needed to assess the differences of the diets in tolerability and efficacy in inducing adequate GMS.

Our study has some limitations. First, we did not give heparin to patients with BHB levels higher than 0.35 mmol/l. Thus, we cannot evaluate the effect of heparin in those with an elevated BHB level as a sign of successful preparation. However, our study focused specifically on the potential value of the point-of-care BHB measurement to guide the use of heparin. In this study setting, it was therefore not appropriate to give heparin to all subjects. Second, we did not randomize patients with low BHB level to receive or not to receive heparin. According to our results, the heparin and no-heparin groups were basically very similar in their basic characteristics. Therefore, our study setting also seems valid. Third, we did not measure post-heparin BHB level and thus cannot analyze the effect of heparin on the BHB level as such.

In our study, patients with obesity tended to have higher probability of adequate suppression even with a low BHB level. More detailed research into the effect of fat deposits on myocardial metabolism might be warranted.

## Conclusions

In summary, we aimed to assess whether BHB measurement could be used to guide heparin use in cardiac [^18^F]FDG-PET/CT. We conclude that BHB level is a good predictor of adequate GMS, and heparin does not improve GMS in patients with a low BHB level. Thus, the use of BHB measurement to guide the use of heparin does not seem to lead to the desired result. A large proportion of patients have low BHB level after one day diet. Research into whether longer diet could be helpful to enhance the BHB response as well as to improve GMS is warranted.

## Data Availability

The data underlying this article cannot be shared publicly due to the privacy of individuals who participated in the study. However, the statistical analysis output data will be shared upon request to the corresponding author.

## Abbreviations

BHB: β-hydroxybutyrate
BMI: Body mass index
FDG: Fluorodeoxyglucose
GMS: Glucose metabolism suppression
IU: International units
kg: Kilogram(s)
l: Litre
m^2^: Metre squared
mmol: Millimole(s)
PET/CT: Positron emission tomography with computed tomography
PSF: Point spread function
ROC: Receiver operating characteristics
ROI: Region of interest
SUL_LV_: Left ventricular mean SUL_max_
SUL_max_: Maximum standardized uptake value normalized by calculated lean body mass
SULratio: SUL_LV_ ratio to left ventricular blood pool background
TOF: Time-of-flight
